# Vitrification altered αV and β3 integrin expression in mouse oocytes in a maturation stage-dependent manner

**DOI:** 10.1007/s11033-026-12338-0

**Published:** 2026-07-10

**Authors:** Sahar Andi, Farzad Rajaei, Shahram Darabi

**Affiliations:** 1 Cellular and Molecular Research Center, Research Institute for Prevention of Non-Communicable Diseases, Qazvin University of Medical Sciences, Qazvin, Iran; 2 Department of Anatomy and Physiology, School of Dentistry and Medical Sciences, Charles Sturt University, Orange, NSW Australia

**Keywords:** Vitrification, Survival rate, Integrin αV, Integrin β3, Cryotop, Mouse oocyte

## Abstract

**Objective:**

Integrin αVβ3 has been implicated in oocyte membrane organization and sperm–oocyte interaction. This study investigated the effects of vitrification on post-warming survival and on the surface expression of integrin αV and β3 subunits in mouse oocytes at the germinal vesicle (GV) and metaphase II (MII) stages.

**Materials and methods:**

A total of 400 oocytes were collected from NMRI mice and allocated into four equal groups: non-vitrified GV, vitrified GV, non-vitrified MII, and vitrified MII (*n* = 100 per group). Vitrification was performed using the Cryotop method, followed by one month of storage in liquid nitrogen at − 196 °C. Post-warming survival was assessed morphologically. Integrin αV and β3 expression was evaluated at the protein level by immunocytochemistry and at the transcript level by quantitative real-time reverse-transcription PCR (RT-qPCR).

**Results:**

Vitrification did not significantly affect post-warming survival rates in GV or MII oocytes compared with their non-vitrified counterparts (90% vs. 88%, *P* > 0.05). Immunocytochemical analysis demonstrated that both αV and β3 surface protein expression was significantly higher in MII oocytes than in GV oocytes, irrespective of vitrification status. Vitrification significantly reduced αV and β3 protein expression in MII oocytes compared with non-vitrified MII oocytes (*P* < 0.05 and *P* < 0.001, respectively), whereas reductions in GV oocytes were not statistically significant (*P* > 0.05). Quantitative RT-PCR broadly corroborated these findings: non-vitrified MII oocytes exhibited the highest transcript levels for both subunits, and β3 mRNA was significantly decreased in vitrified MII oocytes (*P* < 0.05). Although αV transcript levels showed a downward trend following vitrification, these reductions did not reach statistical significance in either stage (*P* > 0.05).

**Conclusion:**

Despite preserving post-warming morphological survival, vitrification induced significant alterations in αV and β3 integrin expression in a maturation stage-dependent manner, with MII oocytes being disproportionately affected. These findings suggest that molecular analysis of membrane-associated proteins may provide information complementary to morphological criteria when assessing oocyte quality following cryopreservation.

## Introduction

Recent advances in reproductive cryobiology have enabled the effective preservation of female germ cells, offering critical fertility options for patients facing conditions such as cancer, endometriosis, polycystic ovary syndrome (PCOS), ovarian hyperstimulation syndrome (OHSS), or failed in vitro fertilization (IVF), particularly when spermatozoa are unavailable at the time of fertilization [1]. In specific circumstances, including prepubertal patients and those with certain chromosomal disorders, immature oocyte cryopreservation represents the preferred, and sometimes the only feasible, clinical approach [2]. Vitrification is currently regarded as the gold standard for oocyte cryopreservation owing to its superior post-warming survival rates and reproductive outcomes relative to conventional slow freezing [3, 4]. This ultrarapid cooling process solidifies a concentrated cryoprotectant solution into a glass-like amorphous state, thereby minimizing intracellular ice crystal formation while better preserving cellular ultrastructure. Nevertheless, vitrification carries inherent risks; cryoprotectant toxicity, osmotic stress, cold shock, and incomplete vitrification can collectively compromise oocyte quality [5]. These insults may induce structural alterations including zona pellucida (ZP) hardening, meiotic spindle disruption, and plasma membrane modification, all of which can impair fertilization competence [5].

Increasing evidence indicates that environmental and in vitro stressors can compromise oocyte competence by disrupting cytoskeletal organization, cell–matrix interactions, and gene expression across several mammalian species [6, 7]. These observations support the hypothesis that vitrification-induced stress may influence oocyte quality through molecular alterations in integrin pathways, providing a clear rationale for investigating receptor-level vulnerabilities following cryopreservation.

Integrins are heterodimeric transmembrane glycoproteins composed of non-covalently associated α and β subunits that mediate cell–cell and cell–extracellular matrix interactions [8]. Beyond their adhesive functions, integrins regulate critical intracellular signalling pathways involving calcium mobilization, pH regulation, cytoskeletal reorganization, and tyrosine phosphorylation cascades [9]. Multiple integrin subunits have been identified on unfertilized oocytes across various species, suggesting that their involvement in oocyte biology extends beyond simple structural roles [10]. Although the αVβ3 heterodimer has been detected on spermatozoa with a proposed contribution to gamete interaction [11], its surface expression on oocytes at different meiotic stages has not been fully characterized. Given that integrin expression at the plasma membrane is associated with membrane integrity and molecular organization [8], characterizing αVβ3 on the oocyte surface may yield insights into these aspects of oocyte quality. Vitrification has been shown to alter integrin gene expression in mouse oocytes [12]. Understanding its differential effects at distinct meiotic stages remains an important question. Therefore, the present study aimed, for the first time, to characterize αV and β3 integrin expression in mouse GV- and MII-stage oocytes and to evaluate the stage-dependent effects of vitrification on post-warming survival and integrin expression using RT-qPCR and immunocytochemistry.

## Materials and methods

### Animals

Adult NMRI female mice were maintained under standard laboratory conditions (12 h light/12 h dark cycle, 22 ± 2 °C) with ad libitum access to food and water.

## Superovulation induction and oocyte collection

Superovulation was induced by intraperitoneal injection of 10 IU pregnant mare serum gonadotropin (PMSG; Folligon, Intervet, Australia), followed 48 h later by 10 IU human chorionic gonadotropin (hCG; Serono, Switzerland). For collection of GV-stage oocytes, mice (*n* = 15) were sacrificed by cervical dislocation 48 h after PMSG administration. Ovaries were excised, placed in flushing medium (Origio, Denmark), and antral follicles were punctured with insulin needles under a stereomicroscope to release cumulus–oocyte complexes. For MII-stage oocytes, hCG was administered 48 h after PMSG injection and mice (*n* = 15) were sacrificed 12–16 h later. Fallopian tubes were isolated and cumulus–oocyte complexes were obtained by tearing the ampullary region. Cumulus and corona radiata cells were removed enzymatically using ICSI Cumulase (Origio, Denmark) and, where necessary, by gentle mechanical pipetting [13]. Only oocytes exhibiting normal morphology (clear cytoplasm, intact ZP, and uniform perivitelline space) were selected for downstream experiments.

## Experimental design

A total of 400 oocytes were distributed across four experimental groups based on maturation stage and vitrification status: non-vitrified GV (*n* = 100), vitrified GV (*n* = 100), non-vitrified MII (*n* = 100), and vitrified MII (*n* = 100). Following the vitrification–warming cycle, surviving oocytes were subjected to downstream analyses.

Protein expression of αV and β3 integrins was assessed by immunocytochemistry (ICC) across three biological replicates per group. For gene expression analysis, oocytes from each group were pooled in sets of 20, yielding three replicates derived from separate experimental runs. Relative mRNA levels of αV and β3 integrins were quantified by RT-qPCR and normalized to β-actin as the reference gene, with each sample run in technical triplicate.

## Vitrification and warming

GV- and MII-stage oocytes were vitrified using a two-step Cryotop protocol (Origio, Denmark). Oocytes were first equilibrated in dehydration/equilibration solution for 10 min at room temperature and then transferred into vitrification solution (VS). They were loaded onto Cryotop strips (Kitazato, Japan) at a density of 1–3 oocytes per strip within 60 s before being rapidly immersed in liquid nitrogen (LN₂) and stored for one month [14].

For warming, the protective cover of the Cryotop was removed while submerged in LN₂, and the strip was immediately transferred into pre-warmed thawing solution (TS; Origio, Denmark) at 37 °C for 1 min. Oocytes were then sequentially transferred into dilution solution (DS) and two washing solutions (WS1 and WS2) for 3 min each [14]. Following warming, oocytes were cultured for 2 h, after which viability was assessed under an inverted microscope. Oocytes exhibiting spherical morphology, an intact plasma membrane, an undamaged ZP, and homogeneous cytoplasm were classified as viable.

## Immunocytochemical assessment of αV and β3 integrin surface expression

Surface expression of integrin subunits αV and β3 was evaluated by immunocytochemistry. Oocytes were washed three times in phosphate-buffered saline (PBS, Gibco, Grand Island, NY, USA), and the zona pellucida was removed by brief exposure to acid Tyrode’s solution for 30 s, followed by extensive PBS washing. Zona-free oocytes were fixed in 3.7% paraformaldehyde (Wako, Japan) for 20 min at room temperature, washed in PBS, and blocked with 5% normal serum in PBS for 1 h to reduce non-specific binding.

To restrict detection to membrane-associated integrin pools, no permeabilization step was performed prior to immunostaining. Oocytes were incubated simultaneously with primary antibodies against integrin αV (1:100; orb13515, Biorbyt, Cambridge, UK; rabbit polyclonal) and integrin β3 (1:100; ab9509, Abcam, Cambridge, UK; mouse monoclonal, clone Y2/51) overnight at 4 °C. Following three PBS washes, oocytes were incubated simultaneously with two species-specific fluorescent secondary antibodies: Alexa Fluor 488-conjugated anti-rabbit IgG (1:500; green channel, αV) and Alexa Fluor 594-conjugated anti-mouse IgG (1:500; red channel, β3) for 1–2 h at room temperature in the dark. Negative control oocytes were processed identically with primary antibodies omitted to confirm staining specificity.

Following completion of integrin immunostaining, oocytes were briefly permeabilized with 0.1% Triton X-100 (Sigma-Aldrich, Germany) in PBS for 5 min and counterstained with DAPI (4ʹ,6-diamidino-2-phenylindole; 1 µg/mL; Sigma-Aldrich, Germany) for 10 min at room temperature, protected from light, for nuclear visualization. Oocytes were then washed three times in PBS before mounting.

Fluorescence images were acquired using an inverted fluorescence microscope (Olympus IX71, Olympus, Japan) at consistent exposure settings across all groups. Three fluorescence channels were captured independently, DAPI (blue, nuclear), Alexa Fluor 488 (green, αV), and Alexa Fluor 594 (red, β3), and merged using ImageJ software (Wayne Rasband, NIH, Maryland, USA) to generate composite three-color RGB images. Co-localization of αV and β3 at the oocyte surface was identified as yellow signal in merged images, representing spatial overlap of the green and red fluorescence channels. Fluorescence intensity was quantified per oocyte within a standardized region of interest circumscribing the oocyte perimeter, normalized to surface area, and expressed as mean fluorescence intensity per oocyte (%).

### Quantitative real-time RT-PCR

Total RNA was extracted from pools of 20 oocytes per replicate using an RNA extraction kit (Roche Biochemicals, Germany) according to the manufacturer’s instructions. Genomic DNA contamination was eliminated by DNase I treatment (EN0521; Fermentas, Germany). RNA concentration and purity were assessed spectrophotometrically (Eppendorf, Germany). First-strand cDNA was synthesized from 500 ng of total RNA using a cDNA synthesis kit (Pars Tous Biotech, Iran) with oligo(dT) primers. Gene-specific primers for integrin αV, integrin β3, and the reference gene β-actin were designed using NCBI Primer-BLAST and synthesized by CinnaGen Co. (Iran). Primer sequences and product lengths are presented in Table 1.

RT-qPCR was performed on a LightCycler^®^ 480 system (Roche Diagnostics, Germany) using SYBR Green Master Mix (Applied Biosystems, USA). Each 10 µl reaction contained 5 µl SYBR Green Master Mix, 0.5 µl forward primer (10 µM), 0.5 µl reverse primer (10 µM), 3 µl nuclease-free H₂O, and 1 µl cDNA template. The amplification program consisted of an initial denaturation at 95 °C for 5 min, followed by 40 cycles of 95 °C for 15 s, 60 °C for 15–20 s, and 72 °C for 15–30 s. Melting curve analysis was performed by heating from 65 °C to 95 °C at a ramp rate of 0.3 °C/s to confirm amplification specificity, followed by a final cooling step at 30 °C for 20 s. Relative gene expression was calculated using the 2^(–ΔΔCT) method, with β-actin used as the internal reference gene.

**Table 1:** Primer sequences used for RT-qPCR analysisᵃ

Gene	Accession No.	Primer Sequence (5′–3′)	Product Length
Integrin αV	NM_008402	F: CAAGGTGGCATTTGGGTCAG	128
		R: GGCAGTGGGTGGGGTTTATT	
Integrin β3	NM_016780	F: TTACCACGGATGCCAAGACC	150
		R: CCCCAGAGATGGGTAGTCCA	
β-Actin	NM_007393	F: CCACCATGTACCCAGGCATT	140
		R: CAGCTCAGTAACAGTCCGCC	

### Statistical analysis

All quantitative data are expressed as mean ± standard error of the mean (SEM). Group differences were evaluated by one-way analysis of variance (ANOVA), followed by Tukey’s multiple comparison test for pairwise comparisons. Statistical analyses and graphical representations were performed using SPSS Statistics version 20 (IBM Corp., Armonk, NY, USA) and GraphPad Prism version 8.0 (GraphPad Software, San Diego, CA, USA). Statistical significance was set at *P* < 0.05.

## Results

### Post-warming oocyte survival

**Fig. 1:**
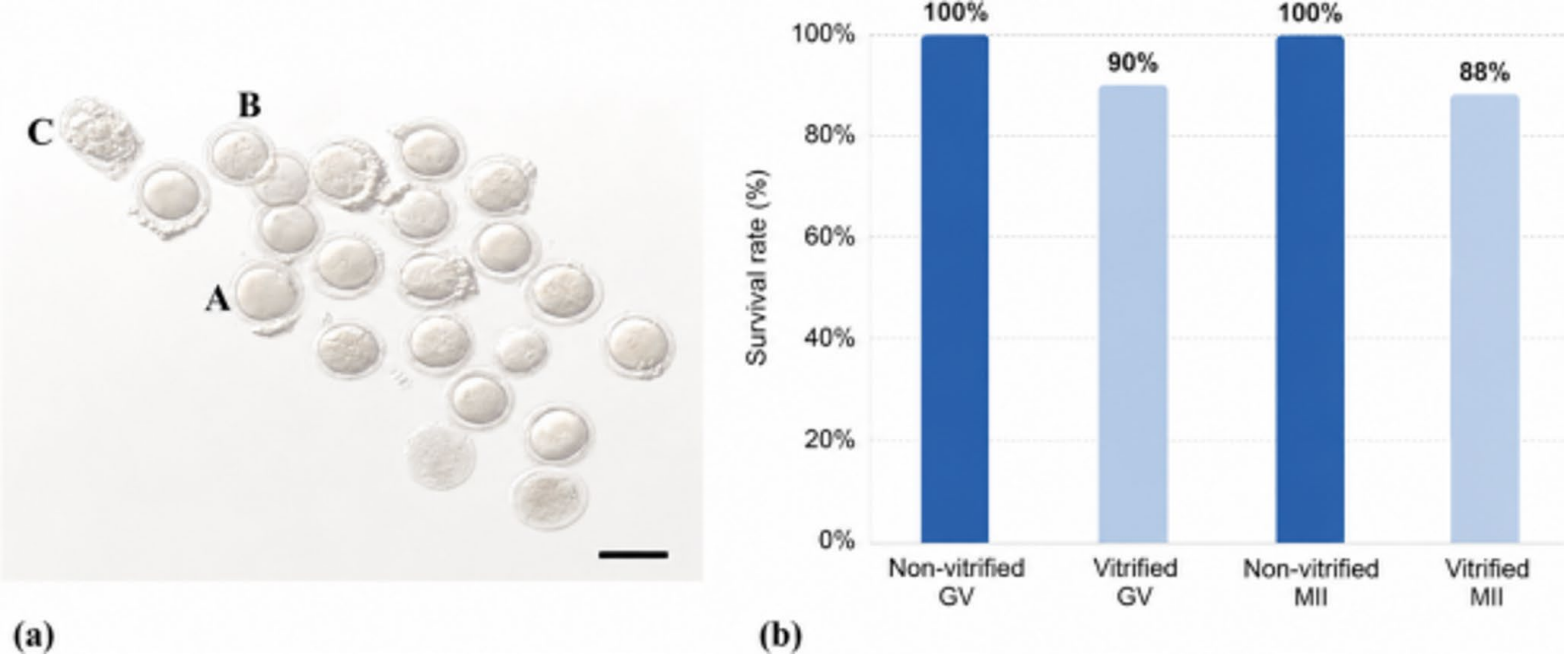
Evaluation of post-vitrification oocyte survival. (a) Representative morphological images of oocytes following warming: intact (A), partially damaged (B), and degenerated (C) oocytes. Scale bar = 100 μm. (b) Post-warming survival rates (%) of GV and MII oocytes in non-vitrified and vitrified groups (mean ± SEM). Vitrification yielded survival rates of 90% (GV) and 88% (MII). No statistically significant difference was observed between vitrified and non-vitrified groups or between GV and MII oocytes within the vitrified groups (*P* > 0.05)

Post-warming survival rates are presented in Fig. 1. Vitrified GV oocytes showed a survival rate of 90%, while vitrified MII oocytes showed a survival rate of 88%. Neither rate differed significantly from the corresponding non-vitrified controls (*P* > 0.05). Furthermore, no significant difference in survival was observed between GV and MII oocytes within the vitrified groups (*P* > 0.05). These results indicate that the Cryotop vitrification protocol effectively preserved oocyte morphological viability irrespective of meiotic stage.

### Immunocytochemical analysis of αV and β3 integrin surface expression

**Fig. 2:**
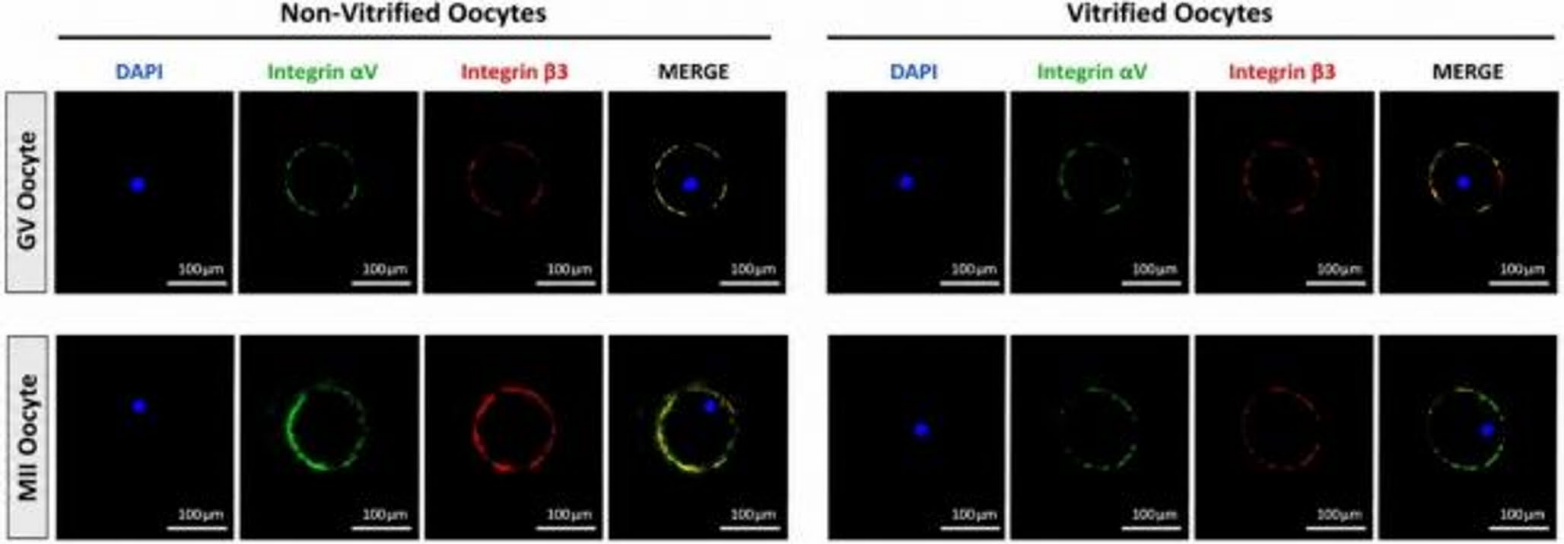
Representative immunofluorescence images showing the surface localization of integrins αV (green) and β3 (red) in zona-free GV- and MII-stage oocytes under non-vitrified and vitrified conditions. Merged images demonstrate co-localization of αV and β3 at the oocyte plasma membrane (yellow). Negative control oocytes, processed without primary antibodies, showed only DAPI-positive nuclei (blue) and no membrane staining, confirming labelling specificity. Integrin expression was higher in MII than in GV oocytes and was reduced by vitrification at both stages, with a significant decrease in vitrified MII oocytes (*P* < 0.05). Scale bar = 100 μm

Immunocytochemical staining of zona-free oocytes revealed distinct surface localization of both αV and β3 integrin subunits. Negative control oocytes exhibited only DAPI nuclear staining and no detectable immunofluorescence signal, confirming antibody specificity. In contrast, experimental groups displayed clear green (αV) and red (β3) fluorescence at the oocyte surface (Fig. 2).

**Fig. 3:**
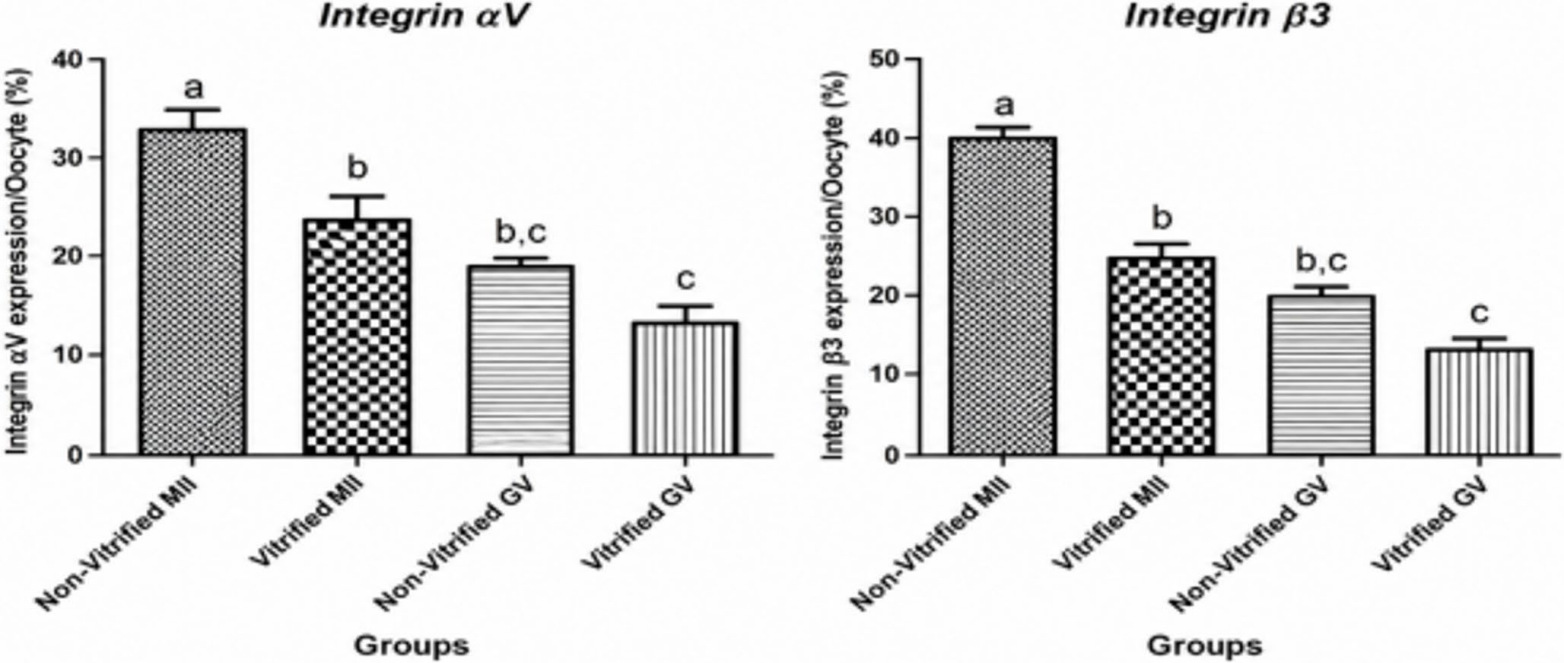
Immunofluorescence quantification of integrin αV and β3 surface expression in non-vitrified and vitrified GV- and MII-stage oocytes. Non-vitrified MII oocytes exhibited the highest staining intensity, while vitrified GV oocytes showed the lowest expression. Vitrification significantly reduced expression in MII oocytes for both αV (*P* < 0.05) and β3 (*P* < 0.001); reductions in GV oocytes did not reach statistical significance (*P* > 0.05). Data are presented as mean ± SEM. Different lowercase letters indicate statistically significant differences among groups (Tukey’s post hoc test, *P* < 0.05)

Integrin αV and β3 surface expression was consistently higher in MII-stage oocytes than in GV-stage oocytes, irrespective of vitrification status (Fig. 3). The highest αV expression was recorded in non-vitrified MII oocytes (32.47 ± 1.91%), and the lowest in vitrified GV oocytes (13.30 ± 1.83%). Vitrification significantly reduced αV surface expression in MII oocytes compared with non-vitrified MII oocytes (22.96 ± 2.74% vs. 32.47 ± 1.91%; *P* < 0.05), whereas the reduction observed in GV oocytes (13.30 ± 1.83 vs.19.34 ± 0.56%) did not attain statistical significance (*P* > 0.05).

Similarly, β3 surface expression was highest in non-vitrified MII oocytes (40.10 ± 1.02%) and lowest in vitrified GV oocytes (12.78 ± 1.40%). Vitrification caused a significant reduction in β3 surface expression in MII oocytes (25.38 ± 2.10% vs. 40.10 ± 1.02%; *P* < 0.001), whereas the reduction in GV oocytes (12.78 ± 1.40 vs. 19.39 ± 1.46%) did not reach statistical significance (*P* > 0.05). Collectively, these findings demonstrate that integrin surface expression is positively associated with oocyte maturation stage and is significantly reduced by vitrification in MII oocytes.

### Quantitative analysis of αV and β3 integrin transcript levels

**Fig. 4:**
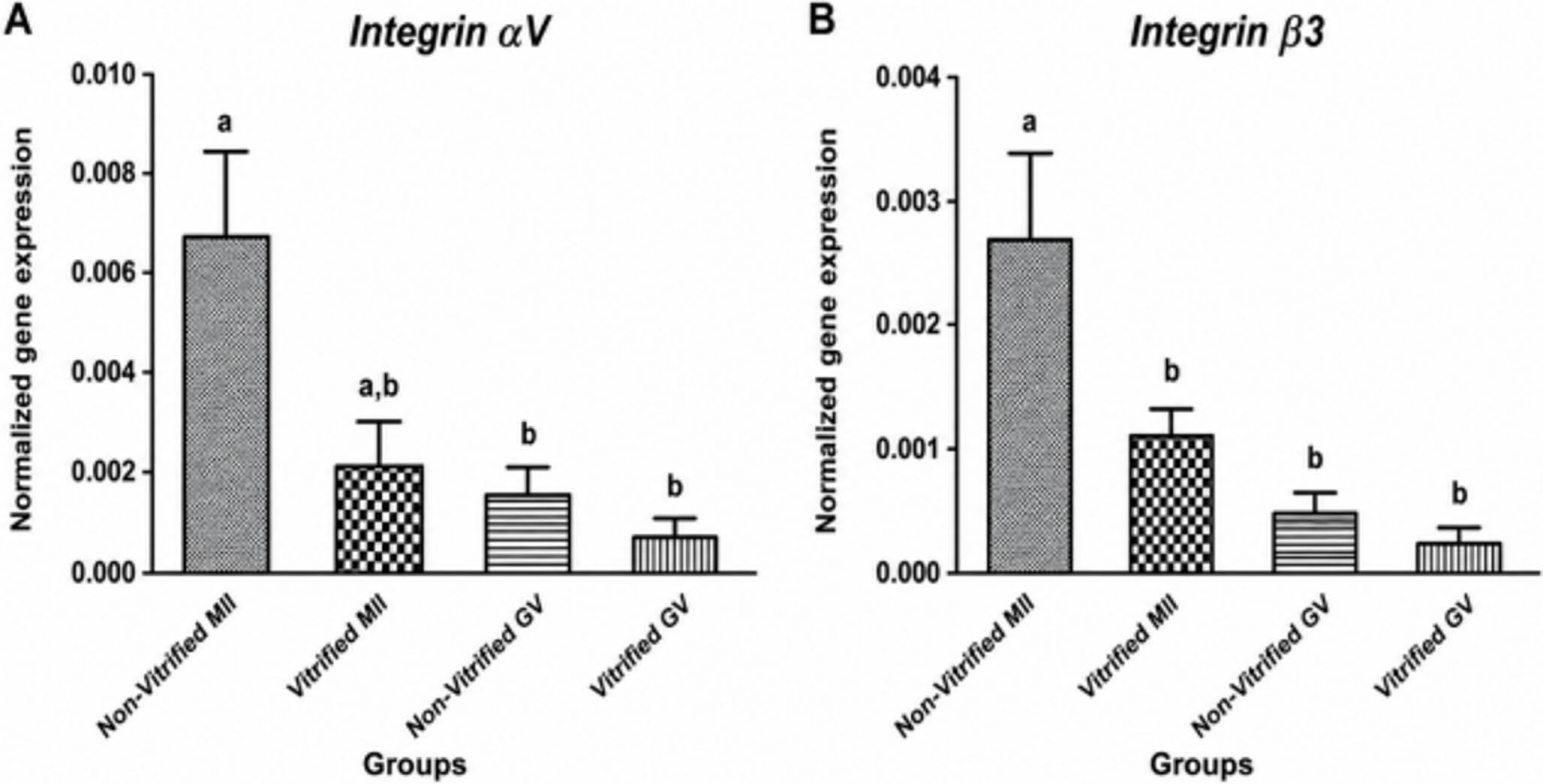
Quantitative real-time RT-PCR analysis of normalized mRNA expression of integrin αV (A) and integrin β3 (B) in non-vitrified and vitrified oocytes at the GV and MII stages. Data are presented as mean ± SEM from three independent experiments. Different lowercase letters (a, b) indicate statistically significant differences among groups (Tukey’s post hoc test, *P* < 0.05)

Quantitative real-time RT-PCR revealed significant differences in αV and β3 integrin mRNA expression among the four experimental groups (αV: *P* = 0.0099; β3: *P* < 0.05; Fig. 4A and B). In both cases, non-vitrified MII oocytes exhibited the highest transcript levels, while vitrified GV oocytes showed the lowest expression.

For integrin αV, vitrification reduced transcript levels in both GV and MII oocytes relative to their non-vitrified counterparts; however, neither reduction achieved statistical significance (*P* > 0.05; Fig. 4A). Overall αV expression was higher in MII than in GV oocytes at both vitrification states, indicating a positive association between αV transcript abundance and oocyte maturation stage.

For integrin β3, vitrification significantly decreased mRNA expression in MII oocytes compared with non-vitrified MII oocytes (*P* < 0.05; Fig. 4B). Although β3 transcript levels were also lower in vitrified GV oocytes than in non-vitrified GV oocytes, this reduction did not reach statistical significance (*P* > 0.05). Taken together, the RT-qPCR results indicate that vitrification exerts a greater negative effect on integrin β3 transcript levels in MII oocytes than in GV oocytes, consistent with the stage-dependent pattern observed at the protein level.

## Discussion

The present study examined the effects of vitrification on post-warming survival and on the expression of αV and β3 integrin subunits at the protein and mRNA levels in GV- and MII-stage mouse oocytes. Vitrification did not significantly affect post-warming survival rates at either the GV (90%) or MII (88%) stage (*P* > 0.05), confirming the effectiveness of the Cryotop protocol irrespective of meiotic stage [15, 16]. These findings are consistent with previous reports demonstrating comparable survival rates between GV and MII oocytes following contemporary vitrification procedures [17, 18]. Nevertheless, conflicting evidence exists; some studies have reported reduced post-warming competence, particularly in MII oocytes, attributed to differences in cryocarrier type, cryoprotectant formulation, and inter-strain biological variability [19–21]. The consistency of survival rates across meiotic stages observed here underscores that morphological post-warming assessment, while necessary, does not fully capture the breadth of cryopreservation-induced cellular alterations. Because sublethal in vitro stressors frequently compromise oocyte quality by destabilizing subcortical scaffolding and cell-matrix interactions without causing overt lysis, a deeper evaluation of surface receptor integrity is fundamentally required. This reality motivated the detailed molecular and transcriptional analyses described herein.

Immunocytochemical analysis revealed distinct surface localization of both αV and β3 integrin subunits on zona-free oocytes, with signal specificity confirmed by the absence of immunoreactivity in negative controls. MII-stage oocytes exhibited markedly higher surface expression of both subunits than GV-stage oocytes, irrespective of vitrification status, consistent with stage-dependent membrane receptor reorganization during meiotic maturation [22, 23]. This upregulation likely reflects the extensive plasma membrane remodelling and cytoskeletal reorganization that accompany meiotic progression and the acquisition of fertilization competence [6, 24].

Vitrification significantly reduced αV and β3 surface expression in MII oocytes (*P* < 0.05 and *P* < 0.001, respectively), whereas reductions in GV oocytes did not attain statistical significance (*P* > 0.05). The greater susceptibility of MII oocytes is consistent with their complex structural organization, including an assembled acentrosomal meiotic spindle, a mature cortical granule layer, and a highly differentiated plasma membrane, all of which are particularly vulnerable to osmotic and thermal stress during cryopreservation [25–27]. In contrast, GV-stage oocytes retain their genetic material within an intact nuclear envelope and lack an assembled spindle, which may confer partial structural protection against cryopreservation-induced membrane perturbation [28]. The significant reduction in integrin surface expression in MII oocytes despite morphologically intact post-warming survival highlights that morphological criteria alone are insufficient to capture the full extent of vitrification-induced molecular changes [29].

A note on integrin detection is warranted for contextual clarity. Earlier studies did not detect αV integrin on the surface of unfertilized mouse oocytes using subunit-specific antibodies [30], whereas the present study identified both αV and β3 subunits at both meiotic stages. This discrepancy most likely reflects differences in antibody clone selection, fixation conditions, epitope accessibility, and detection sensitivity, recognized sources of variability in integrin immunocytochemistry [9, 31]. Supportively, αV integrin has been detected on pig and bovine oocyte plasma membranes by independent laboratories using orthogonal validation approaches [32, 33], indicating that its detectability is subject to species-specific and methodological factors rather than representing a universal biological absence.

Quantitative real-time RT-PCR broadly corroborated the immunocytochemical results. Non-vitrified MII oocytes exhibited the highest transcript levels for both integrin subunits, and a general reduction in expression was observed following vitrification. β3 mRNA expression was significantly decreased in vitrified MII oocytes (*P* < 0.05; Fig. 4B), providing convergent transcriptional evidence for cryopreservation-induced downregulation. In contrast, αV transcript levels showed a consistent downward trend that did not reach statistical significance in either stage (*P* > 0.05; Fig. 4A).

The dissociation between a significant reduction in αV protein expression and the absence of a statistically significant change in αV mRNA likely reflects post-transcriptional and post-translational regulatory mechanisms engaged by cryopreservation stress. Oocytes are transcriptionally quiescent from the late GV stage through fertilization; consequently, protein abundance during this window is governed primarily by translational activation, mRNA stability, and protein turnover rather than de novo transcription [34, 35]. Cryopreservation may therefore reduce αV protein availability through mechanisms such as receptor internalization, impaired vesicular trafficking, or accelerated proteolytic degradation, without producing detectable changes at the mRNA level. Comparable mRNA–protein dissociations have been documented following oocyte vitrification in other molecular contexts [36, 37], underscoring the necessity of evaluating both transcriptional and translational endpoints when assessing the molecular impact of cryopreservation.

Several interconnected mechanisms may underlie the stage-dependent reductions in integrin expression following vitrification. Cryoprotectant-induced osmotic stress can disrupt plasma membrane lipid raft microdomains required for integrin clustering, heterodimer stabilization, and downstream signalling [38, 39]. Concurrently, vitrification elevates intracellular reactive oxygen species (ROS), which impair mitochondrial function, destabilize mRNA species, and promote protein oxidation and degradation [40–42]. Perturbation of the cortical cytoskeleton, which provides the structural scaffolding necessary for integrin membrane anchoring, may further compromise integrin surface density independently of transcriptional effects [43].

Importantly, these findings parallel observations made beyond reproductive biology. In non-gametic systems, stress-induced alterations in integrin-mediated adhesion and signalling have been heavily implicated in cellular injury and recovery across various physiological frameworks. In those settings, the successful restoration of normal baseline homeostasis depends much more fundamentally on the integrity of downstream integrin signalling cascades than on absolute shifts in total integrin abundance alone [6].

Consequently, MII oocytes are likely disproportionately susceptible to vitrification-induced damage owing to their elevated metabolic activity, greater plasma membrane complexity, and strict dependency on an intact, undisturbed cytoskeletal network for spindle stability and cortical organization [6, 24, 26].

## Conclusion

Vitrification altered αV and β3 integrin expression in mouse oocytes in a maturation stage-dependent manner. Significant reductions in surface protein expression were observed in MII-stage oocytes, whereas GV-stage oocytes showed no statistically significant change, despite comparable post-warming survival rates between stages. At the transcript level, β3 mRNA was significantly decreased in vitrified MII oocytes, while αV mRNA changes did not reach statistical significance, indicating that protein and transcriptional responses to cryopreservation stress are not necessarily concordant. These findings suggest that molecular analysis of membrane-associated proteins may offer information complementary to morphological criteria when evaluating oocyte quality following cryopreservation. Further studies employing larger sample sizes and functional endpoints are warranted to elucidate the mechanistic basis of these stage-specific molecular responses and to inform ongoing improvements in oocyte cryopreservation within assisted reproductive technology.

## Data Availability

The data generated and analysed during this study are included in this manuscript. These include oocyte survival rates (GV and MII groups), qRT-PCR gene expression data (αv and β3), immunocytochemistry (ICC) imaging results, statistical analyses between experimental groups, and Cryotop vitrification experimental outcomes. Additional raw data supporting the findings of this study are available from the corresponding author upon reasonable request.
