# Correction: Vitrification altered αV and β3 integrin expression in mouse oocytes in a maturation stage-dependent manner

**DOI:** 10.1007/s11033-026-12463-w

**Published:** 2026-07-27

**Authors:** Sahar Andi, Farzad Rajaei, Shahram Darabi

**Affiliations:** 1 Cellular and Molecular Research Center, Research Institute for Prevention of Non- Communicable Diseases, Qazvin University of Medical Sciences, Qazvin, Iran; 2 Department of Anatomy and Physiology, School of Dentistry and Medical Sciences, Charles Sturt University, Orange, NSW Australia

**Molecular Biology Reports (2026) 53:1132**

10.1007/s11033-026-12338-0

In the original version of this article, the author name Farzad Rajaei was erroneously listed twice in the author group. The correct author group is presented in this erratum.

The original article has been corrected.

